# Risk reduction intervention for raised blood pressure (REVERSE): protocol for a mixed-methods feasibility study

**DOI:** 10.1136/bmjopen-2023-072225

**Published:** 2023-05-31

**Authors:** Lucy Hives, Rachel F Georgiou, Joseph Spencer, Valerio Benedetto, Andrew Clegg, Paul Rutter, Caroline Watkins, Nefyn Williams, Emma P Bray

**Affiliations:** 1 Research Facilitation and Delivery Unit, Applied Health Research Hub, University of Central Lancashire, Preston, UK; 2 Stroke Research Team, School of Nursing, University of Central Lancashire, Preston, UK; 3 Health Technology Assessment Unit, Applied Health Research Hub, University of Central Lancashire, Preston, UK; 4 Methodological Innovation, Development, Adaptation and Support (MIDAS) Theme, NIHR Applied Research Collaboration North West Coast, Liverpool, UK; 5 Faculty of Science and Health, School of Biomedical Sciences, University of Portsmouth, Portsmouth, UK; 6 Stroke Research Team, School of Nursing, Facility of Health and Care, University of Central Lancashire, Preston, UK; 7 Department of Primary Care and Mental Health, Institute of Population Health, University of Liverpool, Liverpool, UK

**Keywords:** Prehypertension, Blood Pressure, Risk Factors, Primary Prevention, Self Care, Feasibility Studies

## Abstract

**Introduction:**

Around 40% of adults have pre-hypertension (blood pressure between 120–139/80–89), meaning they are at increased risk of developing hypertension and other cardiovascular disease-related conditions. There are limited studies on the management of pre-hypertension; however, guidance recommends that it should be focused on lifestyle modification rather than on medication. Self-monitoring of blood pressure could allow people to monitor and manage their risk status and may allow individuals to modify lifestyle factors. The purpose of this study is to determine the feasibility and acceptability, to both healthcare professionals and people with pre-hypertension, of blood pressure self-monitoring.

**Methods and analysis:**

A prospective, non-randomised feasibility study, with a mixed-methods approach will be employed. Eligible participants (n=114) will be recruited from general practices, pharmacies and community providers across Lancashire and South Cumbria. Participants will self-monitor their blood pressure at home for 6 months and will complete questionnaires at three timepoints (baseline, 6 and 12 months). Healthcare professionals and participants involved in the study will be invited to take part in follow-up interviews and a focus group. The primary outcomes include the willingness to engage with the concept of pre-hypertension, the acceptability of self-monitoring, and the study processes. Secondary outcomes will inform the design of a potential future trial. A cost-analysis and cost-benefit analysis will be conducted.

**Ethics and dissemination:**

Ethics approval has been obtained from London–Fulham NHS Research Ethics Committee, the University of Central Lancashire Health Ethics Review Panel and the HRA. The results of the study will be disseminated via peer-reviewed publications, feedback to service users and healthcare professionals, and to professional bodies in primary care and pharmacy.

**Trial registration number:**

ISRCTN13649483.

**STRENGTHS AND LIMITATIONS OF THIS STUDY**

- Recruiting in North West England, which has some of the highest levels of deprivation and worst cardiovascular disease health outcomes in the UK.
- Utilises services from across primary care (general practice, pharmacy and community providers), with the aim of reaching people with pre-hypertension who are likely to be of working age and who might not attend general practice.
- Questionnaire includes clinically validated tools.
- Twelve-month follow-up to allow exploration of longer term outcomes.
- Requirement for individuals to attend a face-to-face appointment for blood pressure eligibility check for those identified through GP electronic records.

## Introduction

People with blood pressure (BP) in the pre-hypertension (PHT) range (120–139/80–89 mm Hg^1^) have an increased risk, compared with those with normal BP, of developing hypertension^2^ and cardiovascular disease (CVD)-related conditions.^3–10^ It is estimated that 40% of adults have BP in the PHT range and this is increasing.^11^

PHT itself is not regarded as a disease, rather it is a warning of progression of BP to problematic levels requiring intervention.^12^ PHT can be a useful sign to alert those at-risk of developing hypertension and CVD, so they can take action to delay or reduce their risk of progression to disease status.^12^ Guidance recommends that PHT management should be focused on lifestyle modification rather than relying on medication as in hypertension.^13^ Prospective cohort studies have shown that by making lifestyle changes, people with PHT can significantly reduce both their risk of developing hypertension^14^ and also reverse PHT to normotensive levels.^15 16^

Evidence from other conditions such as hypertension^17^ and diabetes^18^ shows that self-monitoring (SM) approaches are effective in reducing CVD risk by supporting lifestyle changes. However, unintended negative consequences (UNCs) of such interventions have been reported including anxiety,^19^ poorer self-rated health^20^ and social stigma.^21^ There are also concerns that intervening at the ‘pre-risk’ stage has the effect of labelling, as well as medicalising, the normal.^22^ However, other evidence suggests that making people aware of PHT does not lead to negative effects and may be beneficial.^23 24^

While the technology and procedures involved in SM of BP are already well established in hypertension management, it is premature to assume it will be a feasible, acceptable or effective option for PHT, as the recommended management pathway for the two differ. While a trial to test the effectiveness of SM for PHT is needed, there is currently a scarcity of data to inform its design.^23 24^

The primary aim of REVERSE is to determine the feasibility and acceptability, to both individuals and health care professionals (HCPs), of SM of BP for people with PHT. The results will determine the appropriateness, and design of any future multi-centre, randomised trial to investigate whether SM helps in detecting and increasing awareness of PHT, as well as encouraging lifestyle changes to reduce risk of hypertension and associated CVD.

## Methods and analysis

### Design

The REVERSE study is a prospective, non-randomised feasibility study, using mixed-methods. The study began in October 2021 and will be completed by the end of March 2024.

### Setting

General practices, pharmacies and a community health check provider from across Lancashire and South Cumbria, selected based on Index of Multiple Deprivation (IMD) category (low, mid and high) to ensure socio-economic variation and geographical spread, will act as participant identification sites (PICs).

A search of electronic patient records will be the primary method of identification in general practice. Of those identified as potentially eligible, batches of 50–100 will be randomly chosen and posted a letter of invitation and participant information sheet.

Pharmacies and the community health check provider will identify potential participants when they complete NHS health checks or BP checks. General practice can also opt into this route.

Individuals with a BP reading in the PHT range (second reading 120–139 mm Hg systolic and/or 80–89 mm Hg diastolic) will be offered a participant information sheet and given their health/BP check results summary, including their BP readings, height and weight.

### Participant recruitment

Recruitment for the main study will take place between June 2022 and January 2023. Individuals who have received an information sheet and are interested in taking part will be asked to contact the research team.

As last recorded BP in general practice may have been measured some years previously (especially due to COVID-19), interested individuals will be required to have a BP check, usually completed with the research team, to confirm that their BP is currently in the PHT range.

Individuals identified via a health/BP check in a pharmacy, the community or general practice will have their eligibility confirmed on the initial phone call with a member of the research team, before having an on-line baseline research appointment.

#### Eligibility criteria

Inclusion criteria: aged 18 years and over; BP in the PHT range (120–139/80–89 mmHg).

Exclusion criteria: current or previous diagnosis of hypertension; prescribed anti-hypertension medication; pregnant; life-limiting illness; history of stroke, heart attack or other significant CVD; being unable to understand verbal and written English.

#### Sample size

We aim to recruit and train 114 individuals on the study procedures. Based on an estimated attrition rate of 20%, this will result in 90 participants remaining in the study at the 6-month follow-up.

### Data collection

Data associated with the primary and secondary outcomes listed below, participant demographic and clinical information, and monthly self-monitored BP readings will be collected. The patient-reported data will be collected at baseline, 6 months and 12 months. Additionally, anonymised data will be provided from the PICs to enable description of the overall population that the sample came from.

### Primary outcomes

The primary outcome for this study is to determine whether SM for PHT is feasible and acceptable to both individuals with PHT and to HCPs. This will be assessed by exploring;

#### Willingness to engage with the PHT concept and the SM intervention

Assessed using the number and proportion of services and individuals approached expressing an interest in taking part at the time of recruitment.

#### Acceptability of the intervention and study processes

Explored during service provider and participant interviews and questionnaires, covering: (a) opinions and experiences of the study processes and the intervention; (b) barriers and facilitators to SM; (c) suggested changes and improvements; (d) ease of integration into current practice.

### Secondary outcomes

#### Recruitment rates of participants and service providers

Assessed from the proportion of PICs enrolled and actively identifying potential participants, the time taken to recruit the target sample and the proportion of people who are eligible and who consent to take part.

#### Attrition rates and completeness of data

Measured throughout the recruitment period: proportion of people who attend each study timepoint, number withdrawing definitively and from just the intervention, reasons for withdrawal, proportion of missing data.

#### Protocol adherence

Measured throughout the study, including the number, frequency and timing of self-monitored readings; correct action taken each month; accuracy of recorded readings; reasons for non-adherence.

#### Fidelity to the SM

Measured at 6-month follow-up, competency assessments for research team delivering participant training; number of participants deemed as competent to self-monitor during baseline sessions; proportion of appropriate home readings entered into GP clinical systems after 6-month follow-up.

#### Incidence and impact of any UNCs, healthcare resource use and quality of life

Measured at baseline, 6 months and 12 months, health anxiety assessed using the Health Anxiety Inventory (HAI) short version^25^; depression assessed using the Patient Health Questionnaire (PHQ-9)^26^; healthcare utilisation using a participant questionnaire which will capture the consultations, medications and referrals attributable to the intervention, and quality of life assessed using the EQ-5D-5L.^27^

#### Willingness to pay for a BP machine

Assessed using a willingness-to-pay (WTP) questionnaire at 6 months.^28^

#### Extent self-monitoring encourages lifestyle modification

Assessed using the Simple Lifestyle Indicator Questionnaire (SLIQ) score^29^; risk perception score assessed using an adapted questionnaire^30^; the Determinants of Lifestyle Behaviour Questionnaire (DBLQ)^31^; illness perceptions assessed by score on the Illness Perception Questionnaire–Brief^32^; health locus of control assessed by score on the Multidimensional Health Locus of Control (MHLC).^33^

#### Acceptable content and procedures of a future lifestyle component

Assessed using a participant and service provider focus group to discuss the key elements and design of a potential lifestyle component to work alongside SM.

### Procedure

**Figure 1:**
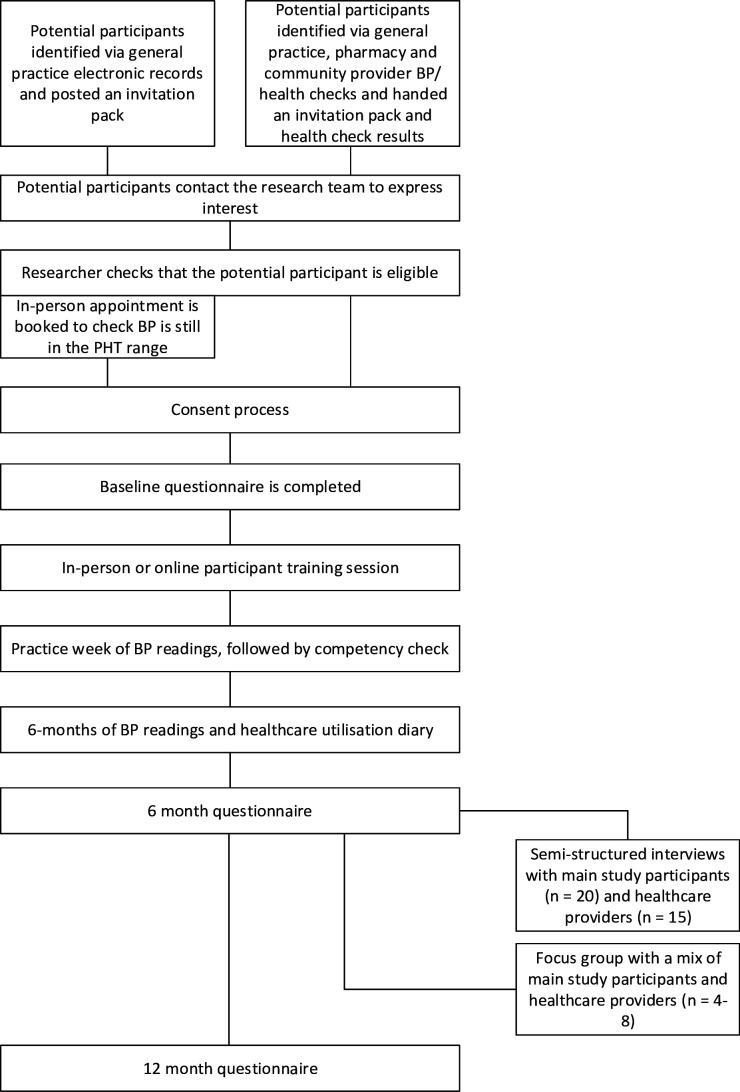
Participant flow through the study.

All participants will take part in BP SM and will proceed through the study as outlined in figure 1.

#### Baseline research appointment

Baseline research clinics will be face to-face for individuals identified from GP patient records and online for those identified via a health/BP check or for those identified from GP records who take their own BP at their practice. At the face-to-face appointment, the research team will take the individuals BP. Eligibility criteria will be checked and consent taken from those eligible and willing to participate. Participants will then complete a questionnaire supported by the researcher. Once completed, a date will be set for the online training and a BP machine and study booklet will be given/posted to each participant.

#### Training in SM of BP and study processes

Participants will be trained by the research team to SM their BP, and this will be supplemented with a training video that will be available on the study website. The 1-hour training session will cover how to SM BP, how to record and send readings to the research team, and what actions to take depending on readings. Following the training session, participants will be asked to complete a practice week to ensure confidence and competency with further training offered if required.

#### SM of BP for 6 months

After their training, each participant will start SM at the beginning of the following month. Participants will be asked to SM their BP for 3 consecutive days, starting on the first Monday of each month, for 6 months. The time of day that readings are taken can be decided by the participant but to minimise confounding factors, the time must be kept consistent throughout the 6 months of SM, ensuring they are done no sooner than 30 min after food or exercise and that the timing of medications in relation to readings is kept the same.

Participants will be asked to record two BP readings, along with the time each reading was taken, on each measurement day. They will colour code the second reading on each day as follows:

- Green for BPs in the normal range (systolic BP (SBP) <115 AND diastolic BP (DBP) <75 mm Hg),
- Amber for those in the PHT range (SBP 115–134 OR DBP 75–84 mm Hg),
- Red for those in the hypertensive range (SBP≥135 OR DBP ≥85 mmHg).

These ranges are based on standard clinical guidelines^1^ but have been reduced by 5/5 mm Hg as recommended for home BP readings. Modified ranges will be provided to relevant participants, for example, the range for those with diabetes will be 10/10 mm Hg lower than guidelines.

After each month’s three measurement days, participants will colour code the overall month as follows:

- If there have been any Red days, the month should be classed as Red,
- If there were no Red days and more Amber than Green days, the month should be Amber,
- If there were no Red days and more Green than Amber days, the month should be classed as Green.

Red, Amber and Green correspond to hypertensive, PHT and normotensive, respectively. If participants' readings are categorised as hypertensive (Red), over 2 consecutive months, they will send their readings to their GP using a covering letter provided by the research team.

Participants will email, post or telephone their readings, colour coding and actions to the research team each month.

#### Six-month follow-up appointment

After 6 months, all participants will be invited to attend a follow-up appointment and complete an evaluation questionnaire. At the appointment, the research team will audit 2 months (randomly selected) of readings stored on participants BP machines for 20% of the sample. The researcher will also support the participant to complete a second study questionnaire.

Participants will be reminded that in a further 6 months’ time, they will be emailed a link (or posted a paper version) to complete a final questionnaire. They will be informed that there is no expectation for them to continue to self-monitor their BP, but they can do so if they wish. Participants will also be invited to express an interest to take part in an interview and/or a focus group (see Qualitative component section below).

With agreement from participants, the research team will contact the relevant general practice of any participant who has had consecutive Red readings to confirm whether the home readings had been received by the practice.

### Data analysis

#### Quantitative analysis

Data will be exported from the Research Electronic Data Capture (REDCap) into SPSS V.27 (IBM Corporation, LLC) and Stata V.16.1 (StataCorp LLC) for analysis. Descriptive analysis will be conducted for the primary and secondary outcomes. For categorical data, outcomes, proportions and percentages will be calculated. For continuous data, mean and SD, or median and IQR will be used. Secondary outcomes will also be analysed for change over time by using summary statistics and exploratory parametric/non-parametric statistical tests.

Subgroup analyses will be undertaken for gender, age range, BP at baseline, index of deprivation and type of provider. The number of categories for these variables will be determined on clinical and statistical grounds. Parameters instrumental to the power calculations required in any future trial will be investigated.

#### Economic analysis

A cost analysis and a cost-benefit analysis will be undertaken. The cost analysis will quantify the health resource utilisation associated with SM. The resource use information will be determined using:

1. Self-reported data at baseline, 6 and 12 months on the participants’ health resource utilisation (eg, medications, consultations and referrals due to their PHT) and will be instrumental in estimating any change pre-study and post-study.
2. Data on human resources (eg, training time) employed to set up the SM obtained from the research team at baseline.

Costs will be quantified by multiplying the resource use expressed in natural units by the unit costs drawn from published sources.^34 35^ SM set-up costs will be calculated in the same way. Where standard costs are not available, costs from published works or expert opinion will be used. The cost-benefit analysis will be based on a WTP using an established approach.^28^

Participants will complete a questionnaire at 6 months that captures key factors relevant to buying a BP machine, the maximum price they would be willing to pay and why. The distribution of the WTP values and how they compare against retail prices for similar BP machines will be determined. Regression analyses will identify potential associations between demographic and clinical variables and the WTP values. As part of the WTP analysis, information about the participants’ health-related quality of life will be collected using the EQ-5D-5L^27^ at baseline, 6 months and 12 months to measure change and estimate Quality Adjusted Life Years (QALYs) from baseline to 6 months and from 6 to 12 months.

### Qualitative component

#### Design and methods

The qualitative components of the study will take place between February and September 2023. Up to 35 (20 main study participants; 15 HCPs (pharmacists, pharmacy assistants, GPs, practice nurses and community practitioners) semi-structured interviews will be conducted, as well as a focus group with a subset of 4–8 individuals. The interview questions will be theory-driven (Theoretical Domains Framework (TDF)^36^) and will explore the acceptability of the SM intervention and study processes, including participant and staff experiences of SM, any barriers and facilitators, and ways the study could be improved in future.

The focus group will explore any links between SM and lifestyle changes, what the content and focus of an intervention should look like and how this should be delivered and operationalised in a future trial.

#### Analysis

Qualitative data will be transcribed and analysed using Framework Analysis^36^ and NVivo software to support analysis. Following data familiarisation, analysis will consist of indexing, charting and mapping, and interpretation. The TDF^37^ will be used as a framework for analysis, but any novel emergent themes will be captured and incorporated through an iterative process of constant comparison. This will identify the experiences of, and barriers to, SM.^38^

### Patient and public involvement

Our two patient and public advisors supported the development of all study materials for participants, including posters, letter of invitation, information sheets, consent forms, questionnaires and training video, and will assist in training participants in BP SM. They will contribute to the analysis and preparation of publications to ensure what is produced is meaningful to patients and the public. If this feasibility study supports proceeding to a full trial, our public advisors will contribute to its design and the co-production of the lifestyle intervention to be tested.

### Ethics and dissemination

A favourable opinion for the study has been received from London–Fulham NHS Research Ethics Committee (REF: 22/PR/0108) and the University of Central Lancashire Health Ethics Review Panel (HEALTH 0299) and from the HRA.

We will publish our findings in open-access journals, through a project website and Twitter feed, and feedback to service users and HCPs at professional conferences and training events, public interest groups and charities.

## Discussion

Prevention of CVD is a major priority for the NHS and the single biggest condition where risk reduction strategies including BP reduction can have an impact.^39^ While there is strong evidence for the effectiveness of SM, both on its own and as a co-intervention in reducing BP in hypertension, there is little known about its role in PHT. REVERSE is the first study to assess the feasibility and acceptability of BP SM for people with PHT. With its mixed-methods design, this study will explore whether individuals are able and willing to engage with the concept of PHT and take action to SM their BP, what individual and HCPs’ experiences of SM are, and allow development of a lifestyle component to implement alongside SM.

The study is being conducted in an area which has the highest levels of deprivation, and worst health outcomes in England, and is an area that could benefit the most from this research. The setting of the study reflects recent service model changes, whereby preventative initiatives are moving away solely from general practice and into pharmacy and communities. Utilising settings across primary care also allows people who do not often visit general practice to be reached. This is especially relevant in people with PHT who may not be symptomatic and who are likely to be of working age.

To lessen the time and cost burden for individuals interested in taking part, research clinics will be conducted online where BP has been taken during the identification process, or where BP needs checking for eligibility, the research team will travel to the individual’s general practice. The study will help general practices identify high BP, a key CVD prevention priority. Due to COVID-19, many people have not had their BP checked for a number of years and so with patient permission, high study BP measurements can be provided to GPs for their records and ongoing management.

A limitation of this study is the research team needing to see people for an eligibility BP check. The original design was for all potential participants to be identified via routine health checks across all providers. However, due to the timing of this study in relation to COVID-19, general practices were not offering regular health checks. This has created an extra step in the recruitment process that otherwise would not have been needed.

The main results of this study, expected in April 2024, will inform future research investigating the effectiveness of SM plus lifestyle interventions, in reducing risk of developing hypertension and cardiovascular conditions.

## Supplementary Material

Reviewer comments

Author's manuscript
