# Supplementary material for: Risk reduction intervention for raised blood pressure (REVERSE): protocol for a mixed-methods feasibility study
Source: BMJ Open. 2023 May 31;13(5):e072225. doi: 10.1136/bmjopen-2023-072225 (PMC10254996; doi:10.1136/bmjopen-2023-072225)
Supplement: Author's manuscript [file bmjopen-2023-072225.draft_revisions.pdf]

BMJ Open is committed to open peer review. As part of this commitment we make the peer review history of every article we publish publicly available.

When an article is published we post the peer reviewers' comments and the authors' responses online. We also post the versions of the paper that were used during peer review. These are the versions that the peer review comments apply to.

The versions of the paper that follow are the versions that were submitted during the peer review process. They are not the versions of record or the final published versions. They should not be cited or distributed as the published version of this manuscript.

BMJ Open is an open access journal and the full, final, typeset and author-corrected version of record of the manuscript is available on our site with no access controls, subscription charges or pay-per-view fees (http://bmjopen.bmj.com).

If you have any questions on BMJ Open's open peer review process please email info.bmjopen@bmj.com

Risk reduction intervention for raised blood pressure (REVERSE): Protocol for a mixed methods feasibility study

Journal:	*BMJ Open*
Manuscript ID	bmjopen-2023-072225
Article Type:	Protocol
Date Submitted by the Author:	25-Jan-2023

Complete List of Authors:	Hives, Lucy; University of Central Lancashire, Research Facilitation and Delivery Unit, Applied Health Research Hub<br>Georgiou, Rachel; University of Central Lancashire, Stroke Research Team, School of Nursing<br>Spencer, Joseph; University of Central Lancashire, Research Facilitation and Delivery Unit, Applied Health Research Hub<br>Benedetto, Valerio; University of Central Lancashire, Health Technology Assessment Unit, Applied Health Research Hub; NIHR Applied Research Collaboration North West Coast, Methodological Innovation, Development, Adaptation and Support (MIDAS) Theme<br>Clegg, Andrew; University of Central Lancashire, Health Technology Assessment Unit, Applied Health Research Hub; NIHR Applied Research Collaboration North West Coast, Methodological Innovation, Development, Adaptation and Support (MIDAS) Theme<br>Rutter, Paul; University of Portsmouth, School of Biomedical Sciences<br>Watkins, Caroline; University of Central Lancashire, Stroke Research Team, School of Nursing, Facility of Health and Care<br>Williams, Nefyn; University of Liverpool, Department of Primary Care and Mental Health, Institute of Population Health<br>Bray, Emma; University of Central Lancashire, Stroke Research Team, School of Nursing, Facility of Health and Care

Keywords:	Prehypertension, Blood Pressure, Risk Factors, Primary Prevention, Self Care, Feasibility Studies

SCHOLARONE™ Manuscripts

TITLE

Risk reduction intervention for raised blood pressure (REVERSE): Protocol for a mixed methods feasibility study

Corresponding author Dr Emma Bray, Stroke Research Team, School of Nursing, University of Central Lancashire, Preston. Ebray@uclan.ac.uk

Authors

Ms Lucy Hives[1], Ms Rachel F Georgiou[2], Mr Joseph Spencer[1], Dr Valerio Benedetto[3,4], Prof Andrew Clegg[3,4], Prof Paul Rutter[5], Prof Caroline Watkins[2], Prof Nefyn Williams[6], Dr Emma Bray[2]

1. Research Facilitation and Delivery Unit, Applied Health Research Hub, University of Lancashire, Preston UK.
2. Stroke Research Team, School of Nursing, Facility of Health and Care, University of Central Lancashire, Preston UK.
3. Health Technology Assessment Unit, Applied Health Research Hub, University of Lancashire, Preston UK.
4. Methodological Innovation, Development, Adaptation and Support (MIDAS) Theme, NIHR Applied Research Collaboration North West Coast (NIHR ARC NWC), Liverpool UK.
5. School of Biomedical Sciences, University of Portsmouth, Portsmouth UK.
6. Department of Primary Care and Mental Health, Institute of Population Health, University of Liverpool, Liverpool UK.

Word count 3,478.

For peer review only

ABSTRACT

Introduction

Around 40% of adults have pre-hypertension (blood pressure between 120-139/80-89), meaning they are at increased risk of developing hypertension and other cardiovascular disease-related conditions. There are limited studies on the management of pre-hypertension, however guidance recommends that it should be focused on lifestyle modification, rather than on medication. Self-monitoring of blood pressure could allow people to monitor and manage their risk status and may allow individuals to modify lifestyle factors. The purpose of this study is to determine the feasibility and acceptability, to both healthcare professionals and people with pre-hypertension, of blood pressure self-monitoring.

Methods and analysis

A prospective, non-randomised feasibility study, with a mixed-methods approach will be employed. Eligible participants (n=114) will be recruited from general practices, pharmacies and community providers across Lancashire and South Cumbria. Participants will self-monitor their blood pressure at home for 6-months and will complete questionnaires at three timepoints (baseline, 6- and 12-months). Healthcare professionals and participants involved in the study will be invited to take part in follow-up interviews and a focus group. The primary outcomes include the willingness to engage with the concept of pre-hypertension, the acceptability of self-monitoring, and the study processes. Secondary outcomes will inform the design of a potential future trial. A cost-analysis and cost-benefit analysis will be conducted.

Ethics and dissemination

Ethics approval has been obtained. The results of the study will be disseminated via peer-reviewed publications, feedback to service users and Health Care Professionals, and to professional bodies in primary care and pharmacy.

Trial registration number ISRCTN13649483

Keywords Prehypertension; Blood Pressure; Risk Factors; Primary Prevention; Self Care; Feasibility Studies

For peer review only

Strengths and limitations of this study

- Recruiting in North West England, which has some of the highest levels of deprivation and worst CVD health outcomes, in the UK.
- Utilises services from across primary care (general practice, pharmacy and community providers), with the aim of reaching people with pre-hypertension who are likely to be of working age and who might not attend general practice.
- Questionnaire includes clinically validated tools.
- 12-month follow up to allow exploration of longer term outcomes.
- Requirement for individuals to attend a face-to-face appointment for blood pressure eligibility check for those identified through GP electronic records.

INTRODUCTION

People with blood pressure (BP) in the pre-hypertension (PHT) range (120-139/80-89 mmHg [1]) have an increased risk, compared to those with normal BP, of developing hypertension [2] and cardiovascular disease (CVD)-related conditions. [3-10] It is estimated that 40% of adults have blood pressure in the PHT range and this is increasing.[11]

PHT itself is not regarded as a disease, rather it is a warning of progression of BP to problematic levels requiring intervention.[12] PHT can be a useful sign to alert those at-risk of developing hypertension and CVD, so they can take action to delay or reduce their risk of progression to disease status.[12] Guidance recommends that PHT management should be focused on lifestyle modification, rather than relying on medication as in hypertension.[13] Prospective cohort studies have shown that by making lifestyle changes, people with PHT can significantly reduce both their risk of developing hypertension [14] and also reverse PHT to normotensive levels. [15,16]

Evidence from other conditions such as hypertension [17] and diabetes [18] shows that SM approaches are effective in reducing CVD risk by supporting lifestyle changes. However, unintended negative consequences (UNCs) of such interventions have been reported including anxiety [19], poorer self-rated health [20] and social stigma [21]. There are also concerns that intervening at the "pre-risk" stage has the effect of labelling, as well as medicalising, the normal. [22] However, other evidence suggests that making people aware of PHT does not lead to negative effects and may be beneficial. [23,24]

While the technology and procedures involved in self-monitoring of blood pressure are already well established in hypertension management, it is premature to assume it will be a feasible, acceptable or effective option for PHT, as the recommended management pathway for the two differ. Whilst a trial to test the effectiveness of SM for PHT is needed, there is currently a scarcity of data to inform its design. [23,24]

The primary aim of REVERSE is to determine the feasibility and acceptability, to both individuals and HCPs , of SM of BP for people with PHT. The results will determine the appropriateness, and design of any future multi-centre, randomised trial to investigate if SM helps in detecting and increasing awareness of PHT, as well as encouraging lifestyle changes to reduce risk of hypertension and associated CVD.

For peer review only

METHODS AND ANALYSIS

Design

The REVERSE study is a prospective, non-randomised feasibility study, using mixed-methods.

Setting

General practices, pharmacies and a community health check provider from across Lancashire and South Cumbria, selected based on Index of Multiple Deprivation (IMD) category (low, mid and high) to ensure socio-economic variation and geographical spread, will act as Participant Identification Sites (PICs).

A search of electronic patient records will be the primary method of identification in General Practice. Of those identified as potentially eligible, batches of 50-100 will be randomly chosen and posted a letter of invitation and participant information sheet.

Pharmacies and the community health check provider will identify potential participants when they complete NHS health checks or blood pressure checks. General Practice can also opt into this route.

Individuals with a BP reading in the PHT range (2nd reading 120-139 mmHg systolic and/or 80-89 mmHg diastolic) will be offered a participant information sheet and given their health/ BP check results summary, including their BP readings, height and weight.

Participant recruitment

Recruitment will take place between June 2022 and January 2023. Individuals who have received an information sheet and are interested in taking part will be asked to contact the research team.

As last recorded BP in general practice may have been done some years previously (especially due to COVID-19), interested individuals will be required to have a BP-check, usually completed with the research team, to confirm that their BP is currently in the PHT range.

Individuals identified via a health/ BP check in a pharmacy, the community, or general practice, will have their eligibility confirmed on the initial phone call with a member of the research team, before having an on-line baseline research appointment.

Eligibility criteria

Inclusion criteria: aged 18 and over; BP in the PHT range (120-139/80-89 mmHg).

Exclusion criteria: current or previous diagnosis of hypertension; prescribed anti-hypertension medication; pregnant; life-limiting illness; history of stroke, heart attack or other significant CVD; being unable to understand verbal and written English.

Sample size

We aim to recruit and train 114 individuals on the study procedures. Based on an estimated attrition rate of 20%, this will result in 90 participants remaining in the study at the 6-month follow-up.

Data Collection

Data associated with the primary and secondary outcomes listed below, participant demographic and clinical information, and monthly self-monitored BP readings will be collected. The patient reported data will be collected at baseline, 6-months and 12-months. Additionally, anonymised data will be provided from the PICs to enable description of the overall population that the sample came from.

Primary outcomes

The primary outcome for this study is to determine whether SM for PHT is feasible and acceptable to both individuals with PHT and to HCPs. This will be assessed by exploring;

Willingness to engage with the PHT concept and the SM intervention

Assessed using the number and proportion of services and individuals approached expressing an interest in taking part at the time of recruitment.

Acceptability of the intervention and study processes

Explored during service provider and participant interviews and questionnaires, covering: a) opinions and experiences of the study processes and the intervention; b) barriers and facilitators to SM; c) suggested changes and improvements; d) ease of integration into current practice.

Secondary outcomes

Recruitment rates of participants and service providers

For peer review only

Assessed from the proportion of PICs enrolled and actively identifying potential participants, the time taken to recruit the target sample and the proportion of people who are eligible and who consent to take part.

Attrition rates and completeness of data

Measured throughout the recruitment period: proportion of people who attend each study timepoint, number withdrawing definitively and from just the intervention, reasons for withdrawal, proportion of missing data.

Protocol adherence

Measured throughout the study, including the number, frequency and timing of self-monitored readings; correct action taken each month; accuracy of recorded readings; reasons for non-adherence.

Fidelity to the SM

Measured at 6-month follow-up, competency assessments for research team delivering participant training; number of participants deemed as competent to self-monitor during baseline sessions; proportion of appropriate home readings entered into GP clinical systems after 6-month follow up.

Incidence and impact of any UNCs, healthcare resource use and quality of life

Measured at Baseline, 6-months and 12-months, health anxiety assessed using the Health Anxiety Inventory (HAI) short version [25]; depression assessed using the Patient Health Questionnaire (PHQ-9) [26]; health care utilisation using a participant questionnaire which will capture the consultations, medications and referrals attributable to the intervention, and quality of life assessed using the EQ-5D-5L. [27]

Willingness to pay for a BP machine

Assessed using a willingness-to-pay questionnaire at 6-months. [28]

Extent self-monitoring encourages lifestyle modification

Assessed using the Simple Lifestyle Indicator Questionnaire (SLIQ) score [29]; risk perception score assessed using an adapted questionnaire [30]; the Determinants of Lifestyle Behaviour Questionnaire (DBLQ) [31]; illness perceptions assessed by score on the Illness Perception Questionnaire – Brief [32]; health locus of control assessed by score on the Multidimensional Health Locus of Control (MHLC) [33].

Acceptable content and procedures of a future lifestyle component

Assessed using a participant and service provider focus group to discuss the key elements and design of a potential lifestyle component to work alongside SM.

Procedure

All participants will take part in BP self-monitoring, and will proceed through the study as outlined in **Figure 1**.

Baseline research appointment

Baseline research clinics will be face to-face for individuals identified from GP patient records, and online for those identified via a health/ BP check or for those identified from GP records who take their own BP at their practice. At the face-to-face appointment the research team will take the individuals BP. Eligibility criteria will be checked and consent taken from those eligible and willing to participate. Participants will then complete a questionnaire supported by the researcher. Once

For peer review only

completed, a date will be set for the online training and a BP machine and study booklet will be given/posted to each participant.

Training in SM of BP and study processes

Participants will be trained by the research team to SM their BP, and this will be supplemented with a training video that will be available on the study website. The 1-hour training session will cover how to SM BP, how to record and send readings to the research team, and what actions to take depending on readings. Following the training session, participants will be asked to complete a practice week to ensure confidence and competency with further training offered if required.

SM of BP for 6 months

After their training, each participant will start SM at the beginning of the following month. Participants will be asked to SM their BP for three consecutive days, starting on the first Monday of each month, for 6-months. The time of day that readings are taken can be decided by the participant but to minimise confounding factors, the time must be kept consistent throughout the 6-months of SM, ensuring they are done no sooner than 30 minutes after food or exercise, and that the timing of medications in relation to readings is kept the same.

Participants will be asked to record two BP readings, along with the time each reading was taken, on each measurement day. They will colour coding the 2nd reading on each day as follows:

- Green for BPs in the normal range (systolic BP (SBP) <115 AND Diastolic BP (DBP) <75 mmHg),
- Amber for those in the PHT range (SBP 115-134 OR DBP 75-84 mmHg),
- Red for those in the hypertensive range (SBP≥135 OR DBP ≥85 mmHg).

These ranges are based on standard clinical guidelines[1] but have been reduced by 5/5mmHg as recommended for home BP readings. Modified ranges will be provided to relevant participants e.g., range for those with diabetes will be 10/10 mmHg lower than guidelines.

After each month's three measurement days, participants will colour code the overall month as follows:

- If there have been any Red days the month should be classed as Red,
- If there were no Red days and more Amber than Green days, the month should be Amber,
- If there were no Red days and more Green than Amber days, the month should be classed as Green.

Red, Amber and Green correspond to hypertensive, PHT and normotensive respectively. If participant's readings are categorised as hypertensive (Red), over two consecutive months, they will send their readings to their GP using a covering letter provided by the research team.

Participants will email, post or telephone their readings, colour coding and actions to the research team each month.

6-month follow-up appointment

After 6-months, all participants will be invited to attend a follow-up appointment and complete an evaluation questionnaire. At the appointment the research team will audit 2-months (randomly selected) of readings stored on participants BP machines for 20% of the sample. The researcher will also support the participant to complete a second study questionnaire.

Participants will be reminded that in a further 6-months' time, they will be emailed a link (or posted a paper version) to complete a final questionnaire. They will be informed that there is no expectation

For peer review only

for them to continue to self-monitor their BP, but they can do so if they wish. Participants will also be invited to express an interest to take part in an interview and/or a focus group (See qualitative section below).

With agreement from participants, the research team will contact the relevant general practice of any participant who has had consecutive RED readings to confirm whether the home readings had been received by the practice.

Data Analysis

Quantitative Analysis

Data will be exported from the Research Electronic Data Capture (REDCap) into SPSS 27 (IBM Corporation, LLC, Armonk, NY, 2020) and Stata 16.1 (StataCorp LLC, College Station, TX, 2020) for analysis. Descriptive analysis will be conducted for the primary and secondary outcomes. For categorical data, outcomes, proportions and percentages will be calculated. For continuous data, mean and standard deviation, or median and interquartile range will be used. Secondary outcomes will also be analysed for change over time by using summary statistics and exploratory parametric/ nonparametric statistical tests.

Sub-group analyses will be undertaken for gender, age range, BP at baseline, index of deprivation and type of provider. The number of categories for these variables will be determined on clinical and statistical grounds. Parameters instrumental to the power calculations required in any future trial will be investigated.

Economic Analysis

A cost analysis and a cost-benefit analysis will be undertaken. The cost analysis will quantify the health resource utilisation associated with SM. The resource use information will be determined using:

a) Self-reported data at baseline, 6- and 12-months on the participants' health resource utilisation (e.g., medications, consultations and referrals due to their PHT) and will be instrumental in estimating any change pre and post study.
b) Data on human resources (e.g., training time) employed to set up the SM obtained from the research team at baseline.

Costs will be quantified by multiplying the resource use expressed in natural units by the unit costs drawn from published sources. [34,35] SM set-up costs will be calculated in the same way. Where standard costs are not available costs from published works or expert opinion will be used. The cost-benefit analysis will be based on a willingness-to-pay (WTP) using an established approach. [28]

Participants will complete a questionnaire at 6-months that captures key factors relevant to buying a BP machine, the maximum price they would be willing to pay and why. The distribution of the WTP values and how they compare against retail prices for similar BP machines will be determined. Regression analyses will identify potential associations between demographic and clinical variables and the WTP values. As part of the WTP analysis, information about the participants' health-related quality of life will be collected using the EQ-5D-5L [27] at baseline, 6-months and 12-months to measure change and estimate QALYs from baseline to 6-months and from 6 to 12-months.

For peer review only

Qualitative component

Design and methods

Up to 35 (20 main study participants; 15 HCPs (pharmacists, pharmacy assistants, GPs, practice nurses, and community practitioners) semi-structured interviews will be conducted, as well as a focus group with a subset of 4-8 individuals. The interview questions will be theory-driven (Theoretical Domains Framework (TDF) [36]) and will explore the acceptability of the SM intervention and study processes, including; participant and staff experiences of SM, any barriers and facilitators, ways the study could be improved in future.

The focus group will explore any links between SM and lifestyle changes, what the content and focus of an intervention should look like and how this should be delivered and operationalised in a future trial.

Analysis

Qualitative data will be transcribed and analysed using Framework Analysis [36] and NVivo software to support analysis. Following data familiarisation, analysis will consist of indexing, charting and mapping, and interpretation. The TDF [37] will be used as a framework for analysis, but any novel emergent themes will be captured and incorporated through an iterative process of constant comparison. This will identify the experiences of, and barriers to, SM. [38]

Patient and public involvement

Our two patient and public advisors supported the development of all study materials for participants, including posters, letter of invitation, information sheets, consent forms, questionnaires, and training materials video, and will assist in training participants in BP SM. They will contribute to the analysis and preparation of publications to ensure what is produced is meaningful to patients and the public. If this feasibility study supports proceeding to a full trial, our public advisors will contribute to its design and the co-production of the lifestyle intervention to be tested.

ETHICS AND DISSEMINATION

A favourable opinion for the study has been received from London – Fulham NHS Research Ethics Committee (REF: 22/PR/0108) and the University of Central Lancashire Health Ethics Review Panel [HEALTH 0299] and from the HRA.

We will publish our findings in open-access journals, through a project website and Twitter feed, and feedback to service users and HCPs at professional conferences and training events, public interest groups and charities.

DISCUSSION

Prevention of cardiovascular disease is a major priority for the NHS and the single biggest condition where risk reduction strategies including BP reduction can have an impact. [39] While there is strong evidence for the effectiveness of SM, both on its own and as a co-intervention in reducing BP in hypertension, there is little known about its role in PHT. REVERSE is the first study to assess the feasibility and acceptability of BP SM for people with PHT. With its mixed-methods design, this study will explore whether individuals are able and willing to engage with the concept of PHT and take action

For peer review only

to SM their BP, what individual and HCPs' experiences of SM are, and allow development of a lifestyle component to implement alongside SM.

The study is being conducted in an area which has the highest levels of deprivation, and worst health outcomes in England, and is an area that could benefit the most from this research. The setting of the study reflects recent service model changes whereby preventative initiatives are moving away solely from general practice and into pharmacy and communities. Utilising settings across primary care also allows people who do not often visit general practice to be reached. This is especially relevant in people with PHT who may not be symptomatic and who are likely to be of working age.

To lessen the time and cost burden for individuals interested in taking part, research clinics will be conducted online where BP has been taken during the identification process, or where BP needs checking for eligibility, the research team will travel to the individual's general practice. The study will help general practices identify high blood pressure, a key CVD prevention priority. Due to COVID-19 many people have not had their BP checked for a number of years and so with patient permission, high study BP measurements can be provided to GPs for their records and ongoing management.

A limitation of this study is the research team needing to see people for an eligibility BP check. The original design was for all potential participants to be identified via routine health checks across all providers. However, due to the timing of this study in relation to COVID-19, general practices were not offering regular health checks. This has created an extra step in the recruitment process that otherwise would not have been needed.

The main results of this study, expected in April 2024, will inform future research investigating the effectiveness of SM plus lifestyle interventions, in reducing risk of developing hypertension and cardiovascular conditions.

Twitter: @ReverseStudy

Website: www.reversestudy.co.uk

ORCID iDs
Lucy Hives https://orcid.org/0000-0003-4125-4034
Rachel Georgiou https://orcid.org/0000-0002-0920-0602
Joseph Spencer https://orcid.org/0000-0003-3723-7629
Valerio Benedetto https://orcid.org/0000-0002-4683-0777
Andrew Clegg https://orcid.org/0000-0001-8938-7819
Paul Rutter https://orcid.org/0000-0003-4106-1515
Caroline Watkins https://orcid.org/0000-0002-9403-3772
Nefyn Williams https://orcid.org/0000-0002-8078-409X
Emma Bray https://orcid.org/0000-0001-9882-3539

Author contributions EB had the original idea with RG, PR, NW, LH, VB, AC and CW, and gained the funding. LH, EB, RG, and JS wrote the first draft, VB provided the statistical and health economic sections. All authors subsequently critically edited the manuscript. EB will be guarantor for the manuscript. All authors read and approved the final manuscript.

Acknowledgements The authors would like to acknowledge the support and input of Kath Gulson and Laura Dunkley from Pharmacy Lancashire in aiding our engagement with Pharmacies, and all our PIC

For peer review only

sites (Queens Square Medical Centre, York Bridge Street General Practice, Cartmel Surgery, Station House Surgery, Prestige Medical Centre, Warburtons Pharmacy, Grange Pharmacy, Cleveleys Pharmacy, and Choose Health) for identifying potential participants. We would also like to acknowledge the support of Paul Heyworth and Patrick Doherty our patient representatives who have served on the steering board and ensure we make patient-centred decisions. Finally, this work would not be possible without the support of the participants.

Funding statement This work was supported by the NIHR Research for Patent Benefit Programme grant number NIHR201028. The views expressed are those of the author(s) and not necessarily those of the NIHR or the Department of Health and Social Care.

Disclaimer The sponsor and funder had no role in the study design, writing of the paper; or the decision to submit this protocol for publication, which was made jointly by the authors who have all approved the final manuscript.

Competing interests statement NHW is a GP principal in Plas Menai Health Centre, Llanfairfechan, LL33 0PE. He is deputy chair of the NIHR HTA Programme funding committee (commissioned research), and Chief Investigator of two NIHR HTA Programme funded trials. VB, AC and CW are funded by the National Institute for Health and Care Research (NIHR) Applied Research Collaboration North West Coast. The views expressed are those of the authors and not necessarily those of the NHS, the NIHR or the Department of Health and Social Care.

REFERENCES

1 Chobanian AV, Bakris GL, Black HR, *et al*. The seventh report of the Joint National Committee on prevention, detection, evaluation, and treatment of high blood pressure: the JNC 7 report. *JAMA* 2003;289:2560–2572. doi: 10.1001/jama.289.19.2560

2 Wang Q, Wang Z, Yao W, *et al*. Anthropometric indices predict the development of hypertension in normotensive and pre- hypertensive middle-aged women in Tianjin, China: A prospective cohort study. *Medical Science Monitor* 2018;24:1871-9. doi: 10.12659/msm.908257

3 Zheng L, Sun Z, Zhang X, *et al*. Predictors of progression from pre-hypertension to hypertension among rural Chinese adults: Results from Liaoning Province. *European Journal of Cardiovascular Prevention and Rehabilitation* 2010;17(2):217-22. doi: 10.1097/HJR.0b013e328334f417

4 Huang Y, Su L, Cai X, *et al*. Association of all-cause and cardiovascular mortality with pre-hypertension: A meta-analysis. *American Heart Journal* 2014;167(2):160-8.e1. doi: 10.1016/j.ahj.2013.10.023

5 Guo X, Yingxian S. Quantitative association between pre-hypertension and different types of cardiovascular outcomes and diabetes: A systematic review and meta-analysis of prospective studies worldwide. *Heart* 2013;3:A115. doi: 10.1136/heartjnl-2013-304613.311

6 Li Y, Xia P, Xu L, *et al*. A Meta-analysis on pre-hypertension and chronic kidney disease. *PLoS ONE* 2016;11(6):e0156575. doi: 10.1371/journal.pone.0156575

For peer review only

7 Kanno A, Kikuya M, Ohkubo T, *et al*. Pre-hypertension as a significant predictor of chronic kidney disease in a general population: The Ohasama Study. *Nephrology Dialysis Transplantation* 2012;27(8):3218-23. doi: 10.1093/ndt/gfs054

8 Huang Y, Cai X, Liu C, *et al*. Pre-hypertension and the risk of coronary heart disease in Asian and western populations: A meta-analysis. *Journal of the American Heart Association* 2015;4(2):e001519. doi: 10.1161/JAHA.114.001519

9 Lee M, Saver JL, Chang B, *et al*. Baseline pre-hypertension and risk of incident stroke: A meta-analysis. *Stroke* 2011;42(3):e49-e50. doi: 10.1212/WNL.0b013e3182315234

10 Winegarden CR. From "pre-hypertension" to hypertension? Additional evidence. *Annals of Epidemiology* 2005;15(9):720-5. doi: 10.1016/j.annepidem.2005.02.010

11 Joffres M, Falaschetti E, Gillespie C, *et al*. Hypertension prevalence, awareness, treatment and control in national surveys from England, the USA and Canada, and correlation with stroke and ischaemic heart disease mortality: a cross-sectional study. *BMJ Open* 2013;3:e003423. doi: 10.1136/bmjopen-2013-003423

12 Collier SR, Landram MJ. Treatment of pre-hypertension: lifestyle and/or medication. *Vascular Health and Risk Management*. 2012;8:613-19. doi: 10.2147/VHRM.S29138

13 Whelton PK, Carey RM, Aronow WS, *et al*. 2017 ACC/AHA/AAPA/ABC/ACPM/AGS/APhA/ASH/ASPC/NMA/PCNA guideline for the prevention, detection, evaluation, and management of high blood pressure in adults: executive summary: a report of the American College of Cardiology/American Heart Association Task Force on Clinical Practice Guidelines. *Hypertension* 2018;71:e13-e115. doi: 10.1016/j.jacc.2017.11.006

14 Chiu YH, Wu SC, Tseng CD, *et al*. Progression of pre-hypertension, stage 1 and 2 hypertension (JNC 7): a population-based study in Keelung, Taiwan (Keelung Community-based Integrated Screening No. 9). *Journal of Hypertension* 2006;24(5):821-8. doi: 10.1097/01.hjh.0000222750.82820.19

15 Moore LL, Visioni AJ, Qureshi MM, *et al*. Weight loss in overweight adults and the long-term risk of hypertension: The Framingham Study. *Archives of Internal Medicine* 2005;165:1298–303. doi: 10.1001/archinte.165.11.1298

16 Padilla J, Wallace JP, Park S. Accumulation of physical activity reduces blood pressure in pre- and hypertension. *Medicine and Science in Sports and Exercise* 2005;37:1264–75. doi: 10.1249/01.mss.0000175079.23850.95

17 Uhlig K, Patel K, Ip S, *et al*. Self-measured blood pressure monitoring in the management of hypertension : a systematic review and meta-analysis. *Annals of Internal Medicine* 2013;159(3):185-94. doi: 10.7326/0003-4819-159-3-201308060-00008

18 Zhu H, Zhu Y, Leung S. Is self-monitoring of blood glucose effective in improving glycaemic control in type 2 diabetes without insulin treatment: a meta-analysis of randomised controlled trials. *BMJ Open* 2016;6:e010524. doi: 10.1136/bmjopen-2015-010524

19 Hamer M, Batty GD, Stamatakis E, *et al*. Hypertension awareness and psychological distress. *Hypertension* 2010;56(3):547-50. doi: 10.1161/HYPERTENSIONAHA.110.153775

For peer review only

20 Barger SD, Muldoon MF. Hypertension labelling was associated with poorer self-rated health in the Third US National Health and Nutrition Examination Survey. *Journal of Human Hypertension* 2006;20:117-23. doi: 10.1038/sj.jhh.1001950

21 Schabert J, Browne JL, Mosely K, *et al*. Social Stigma in Diabetes. *The Patient- Patient-Centred Outcomes Research* 2013;6(1):1-10. doi: 10.1007/s40271-012-0001-0

22 Sims R, Michaleff ZA, Glasziou P, *et al*. Consequences of a diagnostic label: a systematic scoping review and thematic framework. *Frontiers in Public Health* 2021;9. doi: 10.3389/fpubh.2021.725877

23 Viera A, Lingley K, Esserman D. Effects of labelling patients as pre-hypertensive. *Clinical and Translational Science* 2010; 3(2): S21. doi: 10.3122/jabfm.2010.05.100047

24 Spruill TM, Feltheimer SD, Harlapur M, *et al*. Are there consequences of labelling patients with pre-hypertension? An experimental study of effects on blood pressure and quality of life. *Journal of Psychosomatic Research* 2013;74(5):433-8. doi: 10.1016/j.jpsychores.2013.01.009

25 Salkovskis PM, Rimes KA, Warwick HMC, *et al*. The Health Anxiety Inventory: development and validation of scales for the measurement of health anxiety and hypochondriasis. *Psychological Medicine* 2002;32(5):843-853. doi: 10.1017/s0033291702005822

26 Kroenke K, Spitzer RL, Williams JB. The PHQ-9: validity of a brief depression severity measure. *J Gen Intern Med* 2001;16(9):606-613. doi: 10.1046/j.1525-1497.2001.016009606.x

27 Herdman M, Gudex C, Lloyd A, *et al*. Development and preliminary testing of the new five-level version of EQ-5D (EQ-5D-5L). *Quality of Life Research* 2011; 20(10): 1727-36. doi: 10.1007/s11136-011-9903-x

28 Kaambwa B, Bryan S, Frew E, *et al*; on behalf of the TASMINH2 Investigators. What drives responses to willingness-to-pay questions? A methodological inquiry in the context of hypertension self-management. *Journal of Health Economics and Outcomes Research* 2016;4(2):158-171. doi: 10.36469/9818

29 Godwin M, Streight S, Dyachuk E, *et al*. Testing the Simple Lifestyle Indicator Questionnaire: Initial psychometric study. *Can Fam Physician* 2008;54(1):76-77. PMID: 18208960

30 Kaufman AR, Persoskie A, Twesten J, *et al*. A review of risk perception measurement in tobacco control research. *Tobacco Control* 2020;29:s50-s58. doi: 10.1136/tobaccocontrol-2017-054005

31 Lakerveld J, Bot SDM, Chinapaw MJM, *et al*. Measuring Pathways towards a healthier lifestyle in the Hoorn Prevention Study: the Determinants of Lifestyle Behaviour Questionnaire (DBLQ). *Patient Education and Counselling* 2011;85:e53-58. doi: 10.1016/j.pec.2011.01.014

32 Broadbent E, Petrie KJ, Main J, *et al*. The Brief Illness Perception Questionnaire. *Journal of Psychosomatic Research* 2006;60:631-637. doi: 10.1016/j.jpsychores.2005.10.020

33 Wallston KA, Wallston BS, DeVellis R. Development of the Multidimensional Health Locus of Control (MHLC) Scales. *Health Educ Monogr* 1978;6(2):160-170. doi: 10.1177/109019817800600107

34 Jones K, Burns A. Unit Costs of Health and Social Care 2021. In: *Personal social services research unit*. Canterbury: University of Kent, 2021. doi: 10.22024/UniKent/01.02.92342

For peer review only

35 Joint Formulary Committee. *British National Formulary*. 85th ed. London: BMJ Group and Pharmaceutical Press; 2023.

36 Atkins L, Francis J, Islam R, *et al*. A guide to using the Theoretical Domains Framework of behaviour change to investigate implementation problems. *Implementation Science* 2017;12(77). doi: 10.1186/s13012-017-0605-9

37 Gale NK, Heath G, Cameron E, *et al*. Using the framework method for the analysis of qualitative data in multi-disciplinary health research. *BMC Medical Research Methodology* 2013;13(117). doi: 10.1186/1471-2288-13-117

38 Braun V, Clarke V. Using thematic analysis in psychology. *Qualitative Research in Psychology* 2006;3(2)77-101.

39 NHS. The NHS long term plan [online]. 2019. https://www.longtermplan.nhs.uk/ (accessed 18 January 2023).

FIGURES

Figure 1 Participant flow through the study.

For peer review only

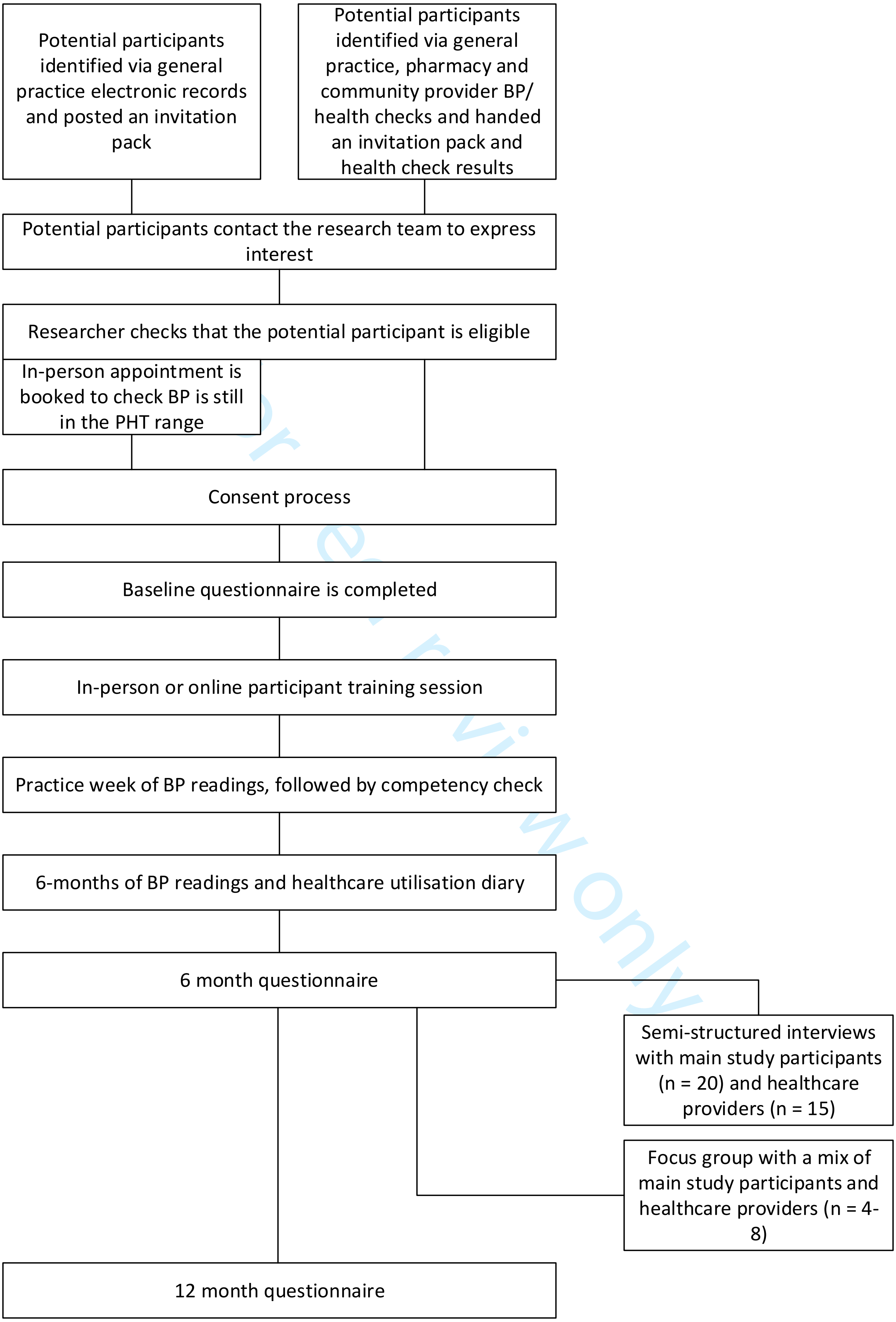

For peer review only

BMJ Open

Risk reduction intervention for raised blood pressure (REVERSE): Protocol for a mixed methods feasibility study

Journal:	*BMJ Open*
Manuscript ID	bmjopen-2023-072225.R1
Article Type:	Protocol
Date Submitted by the Author:	09-Mar-2023
Complete List of Authors:	Hives, Lucy; University of Central Lancashire, Research Facilitation and Delivery Unit, Applied Health Research Hub<br>Georgiou, Rachel; University of Central Lancashire, Stroke Research Team, School of Nursing<br>Spencer, Joseph; University of Central Lancashire, Research Facilitation and Delivery Unit, Applied Health Research Hub<br>Benedetto, Valerio; University of Central Lancashire, Health Technology Assessment Unit, Applied Health Research Hub; NIHR Applied Research Collaboration North West Coast, Methodological Innovation, Development, Adaptation and Support (MIDAS) Theme<br>Clegg, Andrew; University of Central Lancashire, Health Technology Assessment Unit, Applied Health Research Hub; NIHR Applied Research Collaboration North West Coast, Methodological Innovation, Development, Adaptation and Support (MIDAS) Theme<br>Rutter, Paul; University of Portsmouth, School of Biomedical Sciences<br>Watkins, Caroline; University of Central Lancashire, Stroke Research Team, School of Nursing, Facility of Health and Care<br>Williams, Nefyn; University of Liverpool, Department of Primary Care and Mental Health, Institute of Population Health<br>Bray, Emma; University of Central Lancashire, Stroke Research Team, School of Nursing, Facility of Health and Care
**Primary Subject Heading**:	General practice / Family practice
Secondary Subject Heading:	Cardiovascular medicine, Health services research, Public health
Keywords:	Prehypertension, Blood Pressure, Risk Factors, Primary Prevention, Self Care, Feasibility Studies

SCHOLARONE™
Manuscripts

TITLE

Risk reduction intervention for raised blood pressure (REVERSE): Protocol for a mixed methods feasibility study

Corresponding author Dr Emma Bray, Stroke Research Team, School of Nursing, University of Central Lancashire, Preston. Ebray@uclan.ac.uk

Authors

Ms Lucy Hives[1], Ms Rachel F Georgiou[2], Mr Joseph Spencer[1], Dr Valerio Benedetto[3,4], Prof Andrew Clegg[3,4], Prof Paul Rutter[5], Prof Caroline Watkins[2], Prof Nefyn Williams[6], Dr Emma Bray[2]

1. Research Facilitation and Delivery Unit, Applied Health Research Hub, University of Lancashire, Preston UK.
2. Stroke Research Team, School of Nursing, Facility of Health and Care, University of Central Lancashire, Preston UK.
3. Health Technology Assessment Unit, Applied Health Research Hub, University of Lancashire, Preston UK.
4. Methodological Innovation, Development, Adaptation and Support (MIDAS) Theme, NIHR Applied Research Collaboration North West Coast (NIHR ARC NWC), Liverpool UK.
5. School of Biomedical Sciences, University of Portsmouth, Portsmouth UK.
6. Department of Primary Care and Mental Health, Institute of Population Health, University of Liverpool, Liverpool UK.

Word count 3,512.

For peer review only

ABSTRACT

Introduction

Around 40% of adults have pre-hypertension (blood pressure between 120-139/80-89), meaning they are at increased risk of developing hypertension and other cardiovascular disease-related conditions. There are limited studies on the management of pre-hypertension, however guidance recommends that it should be focused on lifestyle modification, rather than on medication. Self-monitoring of blood pressure could allow people to monitor and manage their risk status and may allow individuals to modify lifestyle factors. The purpose of this study is to determine the feasibility and acceptability, to both healthcare professionals and people with pre-hypertension, of blood pressure self-monitoring.

Methods and analysis

A prospective, non-randomised feasibility study, with a mixed-methods approach will be employed. Eligible participants (n=114) will be recruited from general practices, pharmacies and community providers across Lancashire and South Cumbria. Participants will self-monitor their blood pressure at home for 6-months and will complete questionnaires at three timepoints (baseline, 6- and 12-months). Healthcare professionals and participants involved in the study will be invited to take part in follow-up interviews and a focus group. The primary outcomes include the willingness to engage with the concept of pre-hypertension, the acceptability of self-monitoring, and the study processes. Secondary outcomes will inform the design of a potential future trial. A cost-analysis and cost-benefit analysis will be conducted.

Ethics and dissemination

Ethics approval has been obtained from London – Fulham NHS Research Ethics Committee, the University of Central Lancashire Health Ethics Review Panel and the HRA. The results of the study will be disseminated via peer-reviewed publications, feedback to service users and Health Care Professionals, and to professional bodies in primary care and pharmacy.

Trial registration number ISRCTN13649483

Keywords Prehypertension; Blood Pressure; Risk Factors; Primary Prevention; Self Care; Feasibility Studies

Strengths and limitations of this study

- Recruiting in North West England, which has some of the highest levels of deprivation and worst CVD health outcomes, in the UK.
- Utilises services from across primary care (general practice, pharmacy and community providers), with the aim of reaching people with pre-hypertension who are likely to be of working age and who might not attend general practice.
- Questionnaire includes clinically validated tools.
- 12-month follow up to allow exploration of longer term outcomes.
- Requirement for individuals to attend a face-to-face appointment for blood pressure eligibility check for those identified through GP electronic records.

For peer review only

INTRODUCTION

People with blood pressure (BP) in the pre-hypertension (PHT) range (120-139/80-89 mmHg [1]) have an increased risk, compared to those with normal BP, of developing hypertension [2] and cardiovascular disease (CVD)-related conditions. [3-10] It is estimated that 40% of adults have blood pressure in the PHT range and this is increasing.[11]

PHT itself is not regarded as a disease, rather it is a warning of progression of BP to problematic levels requiring intervention.[12] PHT can be a useful sign to alert those at-risk of developing hypertension and CVD, so they can take action to delay or reduce their risk of progression to disease status.[12] Guidance recommends that PHT management should be focused on lifestyle modification, rather than relying on medication as in hypertension.[13] Prospective cohort studies have shown that by making lifestyle changes, people with PHT can significantly reduce both their risk of developing hypertension [14] and also reverse PHT to normotensive levels. [15,16]

Evidence from other conditions such as hypertension [17] and diabetes [18] shows that SM approaches are effective in reducing CVD risk by supporting lifestyle changes. However, unintended negative consequences (UNCs) of such interventions have been reported including anxiety [19], poorer self-rated health [20] and social stigma [21]. There are also concerns that intervening at the “pre-risk” stage has the effect of labelling, as well as medicalising, the normal. [22] However, other evidence suggests that making people aware of PHT does not lead to negative effects and may be beneficial. [23,24]

While the technology and procedures involved in self-monitoring of blood pressure are already well established in hypertension management, it is premature to assume it will be a feasible, acceptable or effective option for PHT, as the recommended management pathway for the two differ. Whilst a trial to test the effectiveness of SM for PHT is needed, there is currently a scarcity of data to inform its design. [23,24]

The primary aim of REVERSE is to determine the feasibility and acceptability, to both individuals and HCPs , of SM of BP for people with PHT. The results will determine the appropriateness, and design of any future multi-centre, randomised trial to investigate if SM helps in detecting and increasing awareness of PHT, as well as encouraging lifestyle changes to reduce risk of hypertension and associated CVD.

METHODS AND ANALYSIS

Design

The REVERSE study is a prospective, non-randomised feasibility study, using mixed-methods. The study began in October 2021 and will be completed by the end of March 2024.

Setting

General practices, pharmacies and a community health check provider from across Lancashire and South Cumbria, selected based on Index of Multiple Deprivation (IMD) category (low, mid and high) to ensure socio-economic variation and geographical spread, will act as Participant Identification Sites (PICs).

A search of electronic patient records will be the primary method of identification in General Practice. Of those identified as potentially eligible, batches of 50-100 will be randomly chosen and posted a letter of invitation and participant information sheet.

For peer review only

Pharmacies and the community health check provider will identify potential participants when they complete NHS health checks or blood pressure checks. General Practice can also opt into this route. Individuals with a BP reading in the PHT range (2nd reading 120-139 mmHg systolic and/or 80-89 mmHg diastolic) will be offered a participant information sheet and given their health/ BP check results summary, including their BP readings, height and weight.

Participant recruitment

Recruitment for the main study will take place between June 2022 and January 2023. Individuals who have received an information sheet and are interested in taking part will be asked to contact the research team.

As last recorded BP in general practice may have been done some years previously (especially due to COVID-19), interested individuals will be required to have a BP-check, usually completed with the research team, to confirm that their BP is currently in the PHT range.

Individuals identified via a health/ BP check in a pharmacy, the community, or general practice, will have their eligibility confirmed on the initial phone call with a member of the research team, before having an on-line baseline research appointment.

Eligibility criteria

Inclusion criteria: aged 18 and over; BP in the PHT range (120-139/80-89 mmHg).

Exclusion criteria: current or previous diagnosis of hypertension; prescribed anti-hypertension medication; pregnant; life-limiting illness; history of stroke, heart attack or other significant CVD; being unable to understand verbal and written English.

Sample size

We aim to recruit and train 114 individuals on the study procedures. Based on an estimated attrition rate of 20%, this will result in 90 participants remaining in the study at the 6-month follow-up.

Data Collection

Data associated with the primary and secondary outcomes listed below, participant demographic and clinical information, and monthly self-monitored BP readings will be collected. The patient reported data will be collected at baseline, 6-months and 12-months. Additionally, anonymised data will be provided from the PICs to enable description of the overall population that the sample came from.

Primary outcomes

The primary outcome for this study is to determine whether SM for PHT is feasible and acceptable to both individuals with PHT and to HCPs. This will be assessed by exploring;

Willingness to engage with the PHT concept and the SM intervention

Assessed using the number and proportion of services and individuals approached expressing an interest in taking part at the time of recruitment.

Acceptability of the intervention and study processes

Explored during service provider and participant interviews and questionnaires, covering: a) opinions and experiences of the study processes and the intervention; b) barriers and facilitators to SM; c) suggested changes and improvements; d) ease of integration into current practice.

Secondary outcomes

For peer review only

Recruitment rates of participants and service providers

Assessed from the proportion of PICs enrolled and actively identifying potential participants, the time taken to recruit the target sample and the proportion of people who are eligible and who consent to take part.

Attrition rates and completeness of data

Measured throughout the recruitment period: proportion of people who attend each study timepoint, number withdrawing definitively and from just the intervention, reasons for withdrawal, proportion of missing data.

Protocol adherence

Measured throughout the study, including the number, frequency and timing of self-monitored readings; correct action taken each month; accuracy of recorded readings; reasons for non-adherence.

Fidelity to the SM

Measured at 6-month follow-up, competency assessments for research team delivering participant training; number of participants deemed as competent to self-monitor during baseline sessions; proportion of appropriate home readings entered into GP clinical systems after 6-month follow up.

Incidence and impact of any UNCs, healthcare resource use and quality of life

Measured at Baseline, 6-months and 12-months, health anxiety assessed using the Health Anxiety Inventory (HAI) short version [25]; depression assessed using the Patient Health Questionnaire (PHQ-9) [26]; health care utilisation using a participant questionnaire which will capture the consultations, medications and referrals attributable to the intervention, and quality of life assessed using the EQ-5D-5L. [27]

Willingness to pay for a BP machine

Assessed using a willingness-to-pay questionnaire at 6-months. [28]

Extent self-monitoring encourages lifestyle modification

Assessed using the Simple Lifestyle Indicator Questionnaire (SLIQ) score [29]; risk perception score assessed using an adapted questionnaire [30]; the Determinants of Lifestyle Behaviour Questionnaire (DBLQ) [31]; illness perceptions assessed by score on the Illness Perception Questionnaire – Brief [32]; health locus of control assessed by score on the Multidimensional Health Locus of Control (MHLC) [33].

Acceptable content and procedures of a future lifestyle component

Assessed using a participant and service provider focus group to discuss the key elements and design of a potential lifestyle component to work alongside SM.

Procedure

All participants will take part in BP self-monitoring, and will proceed through the study as outlined in **Figure 1**.

Baseline research appointment

Baseline research clinics will be face to-face for individuals identified from GP patient records, and online for those identified via a health/ BP check or for those identified from GP records who take their own BP at their practice. At the face-to-face appointment the research team will take the individuals BP. Eligibility criteria will be checked and consent taken from those eligible and willing to

For peer review only

participate. Participants will then complete a questionnaire supported by the researcher. Once completed, a date will be set for the online training and a BP machine and study booklet will be given/posted to each participant.

Training in SM of BP and study processes

Participants will be trained by the research team to SM their BP, and this will be supplemented with a training video that will be available on the study website. The 1-hour training session will cover how to SM BP, how to record and send readings to the research team, and what actions to take depending on readings. Following the training session, participants will be asked to complete a practice week to ensure confidence and competency with further training offered if required.

SM of BP for 6 months

After their training, each participant will start SM at the beginning of the following month. Participants will be asked to SM their BP for three consecutive days, starting on the first Monday of each month, for 6-months. The time of day that readings are taken can be decided by the participant but to minimise confounding factors, the time must be kept consistent throughout the 6-months of SM, ensuring they are done no sooner than 30 minutes after food or exercise, and that the timing of medications in relation to readings is kept the same.

Participants will be asked to record two BP readings, along with the time each reading was taken, on each measurement day. They will colour coding the 2nd reading on each day as follows:

- Green for BPs in the normal range (systolic BP (SBP) <115 AND Diastolic BP (DBP) <75 mmHg),
- Amber for those in the PHT range (SBP 115-134 OR DBP 75-84 mmHg),
- Red for those in the hypertensive range (SBP≥135 OR DBP ≥85 mmHg).

These ranges are based on standard clinical guidelines[1] but have been reduced by 5/5mmHg as recommended for home BP readings. Modified ranges will be provided to relevant participants e.g., range for those with diabetes will be 10/10 mmHg lower than guidelines.

After each month's three measurement days, participants will colour code the overall month as follows:

- If there have been any Red days the month should be classed as Red,
- If there were no Red days and more Amber than Green days, the month should be Amber,
- If there were no Red days and more Green than Amber days, the month should be classed as Green.

Red, Amber and Green correspond to hypertensive, PHT and normotensive respectively. If participant's readings are categorised as hypertensive (Red), over two consecutive months, they will send their readings to their GP using a covering letter provided by the research team.

Participants will email, post or telephone their readings, colour coding and actions to the research team each month.

6-month follow-up appointment

After 6-months, all participants will be invited to attend a follow-up appointment and complete an evaluation questionnaire. At the appointment the research team will audit 2-months (randomly selected) of readings stored on participants BP machines for 20% of the sample. The researcher will also support the participant to complete a second study questionnaire.

For peer review only

Participants will be reminded that in a further 6-months' time, they will be emailed a link (or posted a paper version) to complete a final questionnaire. They will be informed that there is no expectation for them to continue to self-monitor their BP, but they can do so if they wish. Participants will also be invited to express an interest to take part in an interview and/or a focus group (See qualitative section below).

With agreement from participants, the research team will contact the relevant general practice of any participant who has had consecutive RED readings to confirm whether the home readings had been received by the practice.

Data Analysis

Quantitative Analysis

Data will be exported from the Research Electronic Data Capture (REDCap) into SPSS 27 (IBM Corporation, LLC, Armonk, NY, 2020) and Stata 16.1 (StataCorp LLC, College Station, TX, 2020) for analysis. Descriptive analysis will be conducted for the primary and secondary outcomes. For categorical data, outcomes, proportions and percentages will be calculated. For continuous data, mean and standard deviation, or median and interquartile range will be used. Secondary outcomes will also be analysed for change over time by using summary statistics and exploratory parametric/ nonparametric statistical tests.

Sub-group analyses will be undertaken for gender, age range, BP at baseline, index of deprivation and type of provider. The number of categories for these variables will be determined on clinical and statistical grounds. Parameters instrumental to the power calculations required in any future trial will be investigated.

Economic Analysis

A cost analysis and a cost-benefit analysis will be undertaken. The cost analysis will quantify the health resource utilisation associated with SM. The resource use information will be determined using:

a) Self-reported data at baseline, 6- and 12-months on the participants' health resource utilisation (e.g., medications, consultations and referrals due to their PHT) and will be instrumental in estimating any change pre and post study.
b) Data on human resources (e.g., training time) employed to set up the SM obtained from the research team at baseline.

Costs will be quantified by multiplying the resource use expressed in natural units by the unit costs drawn from published sources. [34,35] SM set-up costs will be calculated in the same way. Where standard costs are not available costs from published works or expert opinion will be used. The cost-benefit analysis will be based on a willingness-to-pay (WTP) using an established approach. [28]

Participants will complete a questionnaire at 6-months that captures key factors relevant to buying a BP machine, the maximum price they would be willing to pay and why. The distribution of the WTP values and how they compare against retail prices for similar BP machines will be determined. Regression analyses will identify potential associations between demographic and clinical variables and the WTP values. As part of the WTP analysis, information about the participants' health-related quality of life will be collected using the EQ-5D-5L [27] at baseline, 6-months and 12-months to measure change and estimate QALYs from baseline to 6-months and from 6 to 12-months.

For peer review only

Qualitative component

Design and methods

The qualitative components of the study will take place between February and September 2023. Up to 35 (20 main study participants; 15 HCPs (pharmacists, pharmacy assistants, GPs, practice nurses, and community practitioners) semi-structured interviews will be conducted, as well as a focus group with a subset of 4-8 individuals. The interview questions will be theory-driven (Theoretical Domains Framework (TDF) [36]) and will explore the acceptability of the SM intervention and study processes, including; participant and staff experiences of SM, any barriers and facilitators, ways the study could be improved in future.

The focus group will explore any links between SM and lifestyle changes, what the content and focus of an intervention should look like and how this should be delivered and operationalised in a future trial.

Analysis

Qualitative data will be transcribed and analysed using Framework Analysis [36] and NVivo software to support analysis. Following data familiarisation, analysis will consist of indexing, charting and mapping, and interpretation. The TDF [37] will be used as a framework for analysis, but any novel emergent themes will be captured and incorporated through an iterative process of constant comparison. This will identify the experiences of, and barriers to, SM. [38]

Patient and public involvement

Our two patient and public advisors supported the development of all study materials for participants, including posters, letter of invitation, information sheets, consent forms, questionnaires, and training materials video, and will assist in training participants in BP SM. They will contribute to the analysis and preparation of publications to ensure what is produced is meaningful to patients and the public. If this feasibility study supports proceeding to a full trial, our public advisors will contribute to its design and the co-production of the lifestyle intervention to be tested.

ETHICS AND DISSEMINATION

A favourable opinion for the study has been received from London – Fulham NHS Research Ethics Committee (REF: 22/PR/0108) and the University of Central Lancashire Health Ethics Review Panel [HEALTH 0299] and from the HRA.

We will publish our findings in open-access journals, through a project website and Twitter feed, and feedback to service users and HCPs at professional conferences and training events, public interest groups and charities.

DISCUSSION

Prevention of cardiovascular disease is a major priority for the NHS and the single biggest condition where risk reduction strategies including BP reduction can have an impact. [39] While there is strong evidence for the effectiveness of SM, both on its own and as a co-intervention in reducing BP in hypertension, there is little known about its role in PHT. REVERSE is the first study to assess the feasibility and acceptability of BP SM for people with PHT. With its mixed-methods design, this study

For peer review only

will explore whether individuals are able and willing to engage with the concept of PHT and take action to SM their BP, what individual and HCPs' experiences of SM are, and allow development of a lifestyle component to implement alongside SM.

The study is being conducted in an area which has the highest levels of deprivation, and worst health outcomes in England, and is an area that could benefit the most from this research. The setting of the study reflects recent service model changes whereby preventative initiatives are moving away solely from general practice and into pharmacy and communities. Utilising settings across primary care also allows people who do not often visit general practice to be reached. This is especially relevant in people with PHT who may not be symptomatic and who are likely to be of working age.

To lessen the time and cost burden for individuals interested in taking part, research clinics will be conducted online where BP has been taken during the identification process, or where BP needs checking for eligibility, the research team will travel to the individual's general practice. The study will help general practices identify high blood pressure, a key CVD prevention priority. Due to COVID-19 many people have not had their BP checked for a number of years and so with patient permission, high study BP measurements can be provided to GPs for their records and ongoing management.

A limitation of this study is the research team needing to see people for an eligibility BP check. The original design was for all potential participants to be identified via routine health checks across all providers. However, due to the timing of this study in relation to COVID-19, general practices were not offering regular health checks. This has created an extra step in the recruitment process that otherwise would not have been needed.

The main results of this study, expected in April 2024, will inform future research investigating the effectiveness of SM plus lifestyle interventions, in reducing risk of developing hypertension and cardiovascular conditions.

Twitter: @ReverseStudy

Website: www.reversestudy.co.uk

ORCID iDs
Lucy Hives https://orcid.org/0000-0003-4125-4034
Rachel Georgiou https://orcid.org/0000-0002-0920-0602
Joseph Spencer https://orcid.org/0000-0003-3723-7629
Valerio Benedetto https://orcid.org/0000-0002-4683-0777
Andrew Clegg https://orcid.org/0000-0001-8938-7819
Paul Rutter https://orcid.org/0000-0003-4106-1515
Caroline Watkins https://orcid.org/0000-0002-9403-3772
Nefyn Williams https://orcid.org/0000-0002-8078-409X
Emma Bray https://orcid.org/0000-0001-9882-3539

Author contributions EB had the original idea with RG, PR, NW, LH, VB, AC and CW, and gained the funding. LH, EB, RG, and JS wrote the first draft, VB provided the statistical and health economic sections. All authors subsequently critically edited the manuscript. EB will be guarantor for the manuscript. All authors read and approved the final manuscript.

For peer review only

Acknowledgements The authors would like to acknowledge the support and input of Kath Gulson and Laura Dunkley from Pharmacy Lancashire in aiding our engagement with Pharmacies, and all our PIC sites (Queens Square Medical Centre, York Bridge Street General Practice, Cartmel Surgery, Station House Surgery, Prestige Medical Centre, Warburtons Pharmacy, Grange Pharmacy, Cleveleys Pharmacy, and Choose Health) for identifying potential participants. We would also like to acknowledge the support of Paul Heyworth and Patrick Doherty our patient representatives who have served on the steering board and ensure we make patient-centred decisions. Finally, this work would not be possible without the support of the participants.

Funding statement This work was supported by the NIHR Research for Patent Benefit Programme grant number NIHR201028. The views expressed are those of the author(s) and not necessarily those of the NIHR or the Department of Health and Social Care.

Disclaimer The sponsor and funder had no role in the study design, writing of the paper; or the decision to submit this protocol for publication, which was made jointly by the authors who have all approved the final manuscript.

Competing interests statement NHW is a GP principal in Plas Menai Health Centre, Llanfairfechan, LL33 0PE. He is deputy chair of the NIHR HTA Programme funding committee (commissioned research), and Chief Investigator of two NIHR HTA Programme funded trials. VB, AC and CW are funded by the National Institute for Health and Care Research (NIHR) Applied Research Collaboration North West Coast. The views expressed are those of the authors and not necessarily those of the NHS, the NIHR or the Department of Health and Social Care.

REFERENCES

1 Chobanian AV, Bakris GL, Black HR, *et al*. The seventh report of the Joint National Committee on prevention, detection, evaluation, and treatment of high blood pressure: the JNC 7 report. *JAMA* 2003;289:2560–2572. doi: 10.1001/jama.289.19.2560

2 Wang Q, Wang Z, Yao W, *et al*. Anthropometric indices predict the development of hypertension in normotensive and pre- hypertensive middle-aged women in Tianjin, China: A prospective cohort study. *Medical Science Monitor* 2018;24:1871-9. doi: 10.12659/msm.908257

3 Zheng L, Sun Z, Zhang X, *et al*. Predictors of progression from pre-hypertension to hypertension among rural Chinese adults: Results from Liaoning Province. *European Journal of Cardiovascular Prevention and Rehabilitation* 2010;17(2):217-22. doi: 10.1097/HJR.0b013e328334f417

4 Huang Y, Su L, Cai X, *et al*. Association of all-cause and cardiovascular mortality with pre-hypertension: A meta-analysis. *American Heart Journal* 2014;167(2):160-8.e1. doi: 10.1016/j.ahj.2013.10.023

5 Guo X, Yingxian S. Quantitative association between pre-hypertension and different types of cardiovascular outcomes and diabetes: A systematic review and meta-analysis of prospective studies worldwide. *Heart* 2013;3:A115. doi: 10.1136/heartjnl-2013-304613.311

6 Li Y, Xia P, Xu L, *et al*. A Meta-analysis on pre-hypertension and chronic kidney disease. *PLoS ONE* 2016;11(6):e0156575. doi: 10.1371/journal.pone.0156575

For peer review only

7 Kanno A, Kikuya M, Ohkubo T, *et al*. Pre-hypertension as a significant predictor of chronic kidney disease in a general population: The Ohasama Study. *Nephrology Dialysis Transplantation* 2012;27(8):3218-23. doi: 10.1093/ndt/gfs054

8 Huang Y, Cai X, Liu C, *et al*. Pre-hypertension and the risk of coronary heart disease in Asian and western populations: A meta-analysis. *Journal of the American Heart Association* 2015;4(2):e001519. doi: 10.1161/JAHA.114.001519

9 Lee M, Saver JL, Chang B, *et al*. Baseline pre-hypertension and risk of incident stroke: A meta-analysis. *Stroke* 2011;42(3):e49-e50. doi: 10.1212/WNL.0b013e3182315234

10 Winegarden CR. From "pre-hypertension" to hypertension? Additional evidence. *Annals of Epidemiology* 2005;15(9):720-5. doi: 10.1016/j.annepidem.2005.02.010

11 Joffres M, Falaschetti E, Gillespie C, *et al*. Hypertension prevalence, awareness, treatment and control in national surveys from England, the USA and Canada, and correlation with stroke and ischaemic heart disease mortality: a cross-sectional study. *BMJ Open* 2013;3:e003423. doi: 10.1136/bmjopen-2013-003423

12 Collier SR, Landram MJ. Treatment of pre-hypertension: lifestyle and/or medication. *Vascular Health and Risk Management*. 2012;8:613-19. doi: 10.2147/VHRM.S29138

13 Whelton PK, Carey RM, Aronow WS, *et al*. 2017 ACC/AHA/AAPA/ABC/ACPM/AGS/APhA/ASH/ASPC/NMA/PCNA guideline for the prevention, detection, evaluation, and management of high blood pressure in adults: executive summary: a report of the American College of Cardiology/American Heart Association Task Force on Clinical Practice Guidelines. *Hypertension* 2018;71:e13-e115. doi: 10.1016/j.jacc.2017.11.006

14 Chiu YH, Wu SC, Tseng CD, *et al*. Progression of pre-hypertension, stage 1 and 2 hypertension (JNC 7): a population-based study in Keelung, Taiwan (Keelung Community-based Integrated Screening No. 9). *Journal of Hypertension* 2006;24(5):821-8. doi: 10.1097/01.hjh.0000222750.82820.19

15 Moore LL, Visioni AJ, Qureshi MM, *et al*. Weight loss in overweight adults and the long-term risk of hypertension: The Framingham Study. *Archives of Internal Medicine* 2005;165:1298–303. doi: 10.1001/archinte.165.11.1298

16 Padilla J, Wallace JP, Park S. Accumulation of physical activity reduces blood pressure in pre- and hypertension. *Medicine and Science in Sports and Exercise* 2005;37:1264–75. doi: 10.1249/01.mss.0000175079.23850.95

17 Uhlig K, Patel K, Ip S, *et al*. Self-measured blood pressure monitoring in the management of hypertension : a systematic review and meta-analysis. *Annals of Internal Medicine* 2013;159(3):185-94. doi: 10.7326/0003-4819-159-3-201308060-00008

18 Zhu H, Zhu Y, Leung S. Is self-monitoring of blood glucose effective in improving glycaemic control in type 2 diabetes without insulin treatment: a meta-analysis of randomised controlled trials. *BMJ Open* 2016;6:e010524. doi: 10.1136/bmjopen-2015-010524

19 Hamer M, Batty GD, Stamatakis E, *et al*. Hypertension awareness and psychological distress. *Hypertension* 2010;56(3):547-50. doi: 10.1161/HYPERTENSIONAHA.110.153775

For peer review only

20 Barger SD, Muldoon MF. Hypertension labelling was associated with poorer self-rated health in the Third US National Health and Nutrition Examination Survey. *Journal of Human Hypertension* 2006;20:117-23. doi: 10.1038/sj.jhh.1001950

21 Schabert J, Browne JL, Mosely K, *et al*. Social Stigma in Diabetes. *The Patient- Patient-Centred Outcomes Research* 2013;6(1):1-10. doi: 10.1007/s40271-012-0001-0

22 Sims R, Michaleff ZA, Glasziou P, *et al*. Consequences of a diagnostic label: a systematic scoping review and thematic framework. *Frontiers in Public Health* 2021;9. doi: 10.3389/fpubh.2021.725877

23 Viera A, Lingley K, Esserman D. Effects of labelling patients as pre-hypertensive. *Clinical and Translational Science* 2010; 3(2): S21. doi: 10.3122/jabfm.2010.05.100047

24 Spruill TM, Feltheimer SD, Harlapur M, *et al*. Are there consequences of labelling patients with pre-hypertension? An experimental study of effects on blood pressure and quality of life. *Journal of Psychosomatic Research* 2013;74(5):433-8. doi: 10.1016/j.jpsychores.2013.01.009

25 Salkovskis PM, Rimes KA, Warwick HMC, *et al*. The Health Anxiety Inventory: development and validation of scales for the measurement of health anxiety and hypochondriasis. *Psychological Medicine* 2002;32(5):843-853. doi: 10.1017/s0033291702005822

26 Kroenke K, Spitzer RL, Williams JB. The PHQ-9: validity of a brief depression severity measure. *J Gen Intern Med* 2001;16(9):606-613. doi: 10.1046/j.1525-1497.2001.016009606.x

27 Herdman M, Gudex C, Lloyd A, *et al*. Development and preliminary testing of the new five-level version of EQ-5D (EQ-5D-5L). *Quality of Life Research* 2011; 20(10): 1727-36. doi: 10.1007/s11136-011-9903-x

28 Kaambwa B, Bryan S, Frew E, *et al*; on behalf of the TASMINH2 Investigators. What drives responses to willingness-to-pay questions? A methodological inquiry in the context of hypertension self-management. *Journal of Health Economics and Outcomes Research* 2016;4(2):158-171. doi: 10.36469/9818

29 Godwin M, Streight S, Dyachuk E, *et al*. Testing the Simple Lifestyle Indicator Questionnaire: Initial psychometric study. *Can Fam Physician* 2008;54(1):76-77. PMID: 18208960

30 Kaufman AR, Persoskie A, Twesten J, *et al*. A review of risk perception measurement in tobacco control research. *Tobacco Control* 2020;29:s50-s58. doi: 10.1136/tobaccocontrol-2017-054005

31 Lakerveld J, Bot SDM, Chinapaw MJM, *et al*. Measuring Pathways towards a healthier lifestyle in the Hoorn Prevention Study: the Determinants of Lifestyle Behaviour Questionnaire (DBLQ). *Patient Education and Counselling* 2011;85:e53-58. doi: 10.1016/j.pec.2011.01.014

32 Broadbent E, Petrie KJ, Main J, *et al*. The Brief Illness Perception Questionnaire. *Journal of Psychosomatic Research* 2006;60:631-637. doi: 10.1016/j.jpsychores.2005.10.020

33 Wallston KA, Wallston BS, DeVellis R. Development of the Multidimensional Health Locus of Control (MHLC) Scales. *Health Educ Monogr* 1978;6(2):160-170. doi: 10.1177/109019817800600107

34 Jones K, Burns A. Unit Costs of Health and Social Care 2021. In: *Personal social services research unit*. Canterbury: University of Kent, 2021. doi: 10.22024/UniKent/01.02.92342

For peer review only

35 Joint Formulary Committee. *British National Formulary*. 85th ed. London: BMJ Group and Pharmaceutical Press; 2023.

36 Atkins L, Francis J, Islam R, *et al*. A guide to using the Theoretical Domains Framework of behaviour change to investigate implementation problems. *Implementation Science* 2017;12(77). doi: 10.1186/s13012-017-0605-9

37 Gale NK, Heath G, Cameron E, *et al*. Using the framework method for the analysis of qualitative data in multi-disciplinary health research. *BMC Medical Research Methodology* 2013;13(117). doi: 10.1186/1471-2288-13-117

38 Braun V, Clarke V. Using thematic analysis in psychology. *Qualitative Research in Psychology* 2006;3(2)77-101.

39 NHS. The NHS long term plan [online]. 2019. https://www.longtermplan.nhs.uk/ (accessed 18 January 2023).

FIGURES

Figure 1 Participant flow through the study.

For peer review only

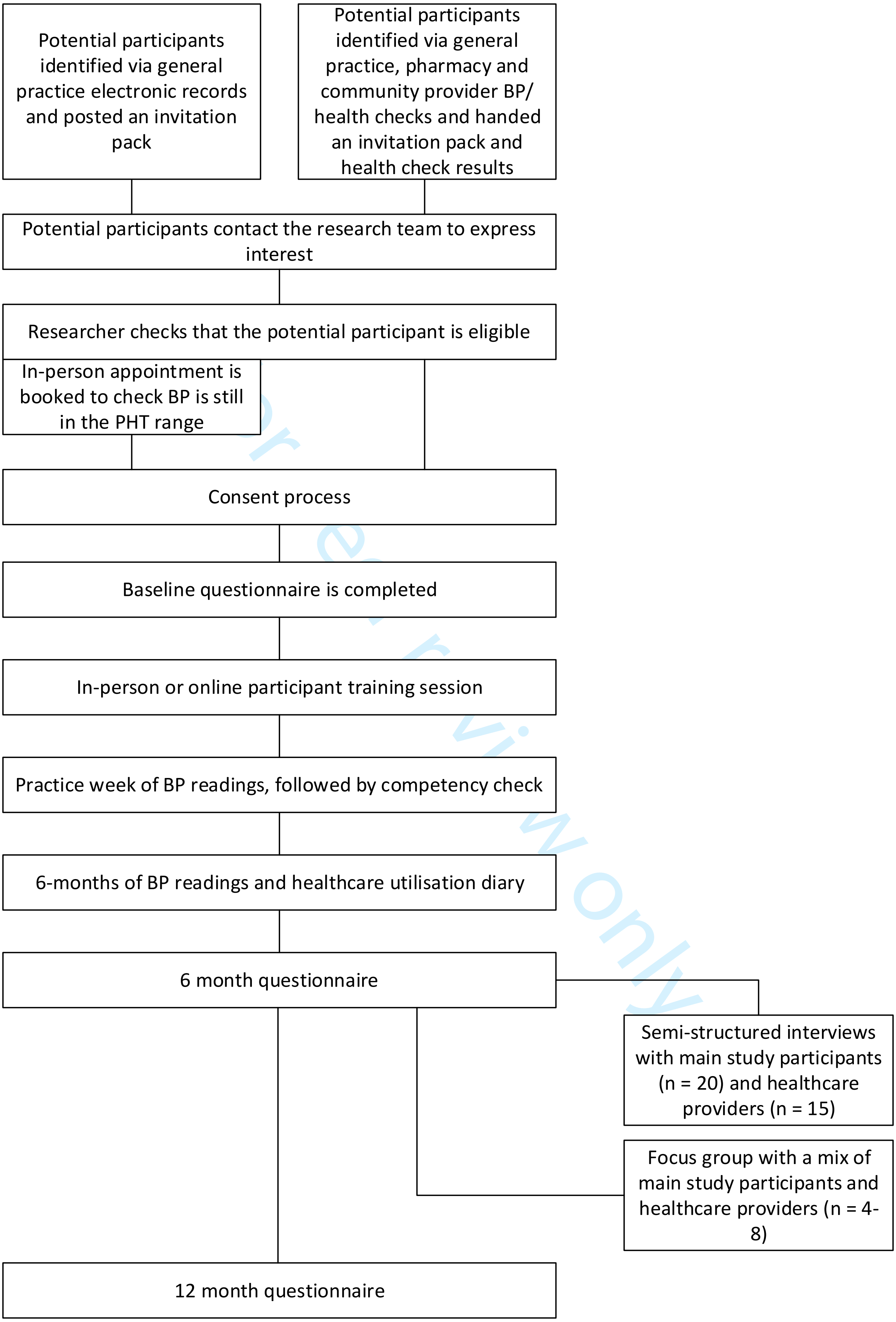
Potential participants identified via general practice electronic records and posted an invitation pack
Potential participants identified via general practice, pharmacy and community provider BP/ health checks and handed an invitation pack and health check results
Potential participants contact the research team to express interest
Researcher checks that the potential participant is eligible
In-person appointment is booked to check BP is still in the PHT range
Consent process
Baseline questionnaire is completed
In-person or online participant training session
Practice week of BP readings, followed by competency check
6-months of BP readings and healthcare utilisation diary
6 month questionnaire
Semi-structured interviews with main study participants (n = 20) and healthcare providers (n = 15)
Focus group with a mix of main study participants and healthcare providers (n = 4-8)
12 month questionnaire
