# Supplementary material for: Risk reduction intervention for raised blood pressure (REVERSE): protocol for a mixed-methods feasibility study
Source: BMJ Open. 2023 May 31;13(5):e072225. doi: 10.1136/bmjopen-2023-072225 (PMC10254996; doi:10.1136/bmjopen-2023-072225)
Supplement: Reviewer comments [file bmjopen-2023-072225.reviewer_comments.pdf]

PEER REVIEW HISTORY

BMJ Open publishes all reviews undertaken for accepted manuscripts. Reviewers are asked to complete a checklist review form (**http://bmjopen.bmj.com/site/about/resources/checklist.pdf**) and are provided with free text boxes to elaborate on their assessment. These free text comments are reproduced below.

ARTICLE DETAILS

**TITLE (PROVISIONAL)**	Risk reduction intervention for raised blood pressure (REVERSE): Protocol for a mixed methods feasibility study
**AUTHORS**	Hives, Lucy; Georgiou, Rachel; Spencer, Joseph; Benedetto, Valerio; Clegg, Andrew; Rutter, Paul; Watkins, Caroline; Williams, Nefyn; Bray, Emma

This article was not externally reviewed at BMJ Open. Protocols that have been independently assessed prior to submission to BMJ Open are usually fast-tracked to publication on the grounds that further substantial changes will not be possible. This independent assessment will usually be external, independent review for both a substantial grant award from a non-commercial or government funder and ethics approval.
